# A Case of Extensive Fracture of the Skull and Rupture of the Meninges, with Loss of the Brain, and Subsequent Recovery

**Published:** 1850-06

**Authors:** John Swain

**Affiliations:** Ballardsville, Ky.


ART. III.—*A Case of extensive Fracture of the Skull and rupture of the meninges, with loss of substance of the brain, and subsequent recovery.* By JOHN SWAIN, M. D., of Ballardsville, Ky.

On the morning of the 13th of October, 1848, after early breakfast, David Forbis, aged 12 years, was found in a pasture, with his skull fractured, by a kick from a horse. Owing to the distance of all medical aid, and to avail themselves of certain necessary surgical assistance, the friends dispatched three messengers, one for Dr. Drane, of New Castle, another for Dr. Baker, of Shelby county, and a third for myself.

I saw the case in one hour and a half after the injury, and was immediately joined by Dr. Baker. We found the patient lying quiet; complained of a little pain; appeared perfectly rational, and answered all questions satisfactorily. His pulse and respiration slow, weak, and irregular; hands and feet cold. We gave him some warm toddy, and applied warmth to his feet. Shortly after taking the toddy he vomited his breakfast, unassimilated, together with a considerable quantity of blood, which had passed from the wound by the posterior nares and the esophagus, to the stomach.

At 10 A. M., we were joined by Dr. Drane, and upon consultation, after a superficial examination, decided, without dissent, that there was but little hope for recovery; but for our own credit, and that of the profession and the satisfaction of the friends, it became our duty to treat the case as we would have treated it, had it presented the most flattering features.

The pulse had now improved a little, yet it could not be said there was positive reaction. We proceeded to make a more particular examination, and to dress the wound.

The blow covered the os frontis and the nasal bones, crushing the former, depressing the latter, separating the head from the face, and, making an external wound, reaching from the outer angle of the right eye, to the lesser cánthus of the left, sufficiently open to admit the passage of the finger behind the ball of either eye.

A portion of the frontal bone of the left side, which is by measurement one inch by one and a half, on its frontal face, including the orbitar arch, and a portion of the orbitar plate, one inch by four lines upon its orbitar surface, was detached from the surrounding tissues, and buried completely in the substance of the brain, displacing about a tablespoonful of the cerebral substance, which, was lost in dressing. Smaller portions of the supra orbitar arch of the right side were raised and removed. The lips of the wound were now drawn together by the interrupted suture, dressed with lint, a compress and common roller.

The examination did not change our opinion first expressed; but confirmed the impression of a fatal termination.

The dressing being accomplished, the patient said he felt quite comfortable. Twice, during the evening, he vomited considerable quantities of blood.

7 P. M. Pulse seventy, soft and regular, respiration twenty, complains of a little pain in his head, has urinated once.

10 P. M. Vomited blood, after which he rested quietly the remainder of the night.

14th,—morning. Pulse seventy-two, respiration normal, feet a little cool, complains of some pain over the left eye, has urinated freely, bowels not moved, prescribed comp. ext. colocynth ʒss., ext. hyoscyami grs. x. Misc. Ft. mass. Div. pill. no. 7. One pill every four hours.

Evening, 8 P. M. Takes note of time by remembering the hours struck; pulse seventy-four, respiration normal, skin normal, excepting feet, which are a little cool, has urinated, bowels not moved, continue pills.

15th,—morning. Has slept tolerably well all night; pulse seventy-four, respiration and skin normal, feet a little cool, tongue has a light, white coat, complains of pain in the head, no operation from bowels; continue pills.

16th. Complains of his head a little; pulse eighty-four, skin hot, tongue still slightly coated, had three small operations from bowels, urinated freely; directed attendants to give a pill at 12 M. and 4 P. M., and to sponge the surface with vinegar and water; left a solution of tart. ant. et pot. to be given if fever increased, and some pulv. Doveri to guard against too free evacuations from bowels.

17th. Slept well most of the night; pulse ninety, respiration twenty, skin a little above normal condition; has taken sol. tart. once; evinces considerable intolerance to light; darkened the room.

18th. Pulse ninety, respiration twenty, tongue as yesterday, bowels moved by medicine, complained of severe pain in a carious tooth, had some pain in the bowels during the night, which gave way upon the application of an emollient poultice, and he slept quietly till morning.

19th. Face swelled, evidently from the decayed tooth, complains a little of his head; pulse eighty-four, respiration nineteen, tongue and skin normal, has urinated freely, no operation from bowels, has taken two pills, directed to take a pill every four hours till bowels are moved—cheerful and humerous.

20th. Rested well last night, complains of but little pain; pulse ninety-two, soft and pleasant, tongue, skin, and respiration normal, urinated freely, bowels not moved.

To-day (8th day of the injury) met Dr. Drane, and removed the dressings for the first time, no swelling about the wound, which presented a healthy appearance; sutures "in situ;" that portion of the wound over the whole of the right eye, and a small portion of the opposite extreme, closed by the first intention; some sloughing, and the discharge of a little healthy matter; dressed the wound with simple cerate, compress and roller. Gave some rice.

21st. Complained of pain in his eyes for an hour after yesterday's dressing, owing no doubt to the amount of light admitted to accomplish it. Rested well all night. Dressed the wound as yesterday, found a free discharge of healthy pus. Pulse one hundred; all other functions normal.

22d. Dressed the wound, pulse ninety, no other change from yesterday.

23d. Pulse eighty-six, no other change; the sutures having given way I removed them and dressed the wound with adhesive straps. The quantity of matter discharged, was very considerable, much more than I had looked for, and continued in quantity up to 30th, when the wound commenced filling with healthy granulations, and daily improved up to December 8, at which time I discontinued my visits.

This patient is now apparently healthy, in the full enjoyment of all his natural faculties and functions, with the solitary exception of the sense of smell, of which, he is wholly deprived. He is not disturbed by the application of aq. ammonia to his nose, says, it feels a little warm, but not unpleasant. He has no recollection of any circumstance connected with the accident; remembers of speaking to a negro in the pasture as he went out, has an indistinct idea of being brought home, but between those two points all is blank.

REMARKS.—In looking at the history of this case we are astonished that the patient should have recovered, and much more so, that there should have been no functional disturbance. The os frontis was so broken up, that it scarcely offered any resistance to pressure, but felt like jelly under the finger; a portion was driven into the brain, and the head and face separated. A blow, so severe and crushing, must have given a terrible shock to the whole brain. Yet, at no time did it suffer in the least, but appeared to be active in all its functions; and every organ moved on in the even tenor of its way. It is true, the bowels demanded some assistance; but would not a man, in health, reqaire as much, if placed and retained in a horizontal position, for the same length of time? Reaction came on quietly and slowly; the pulse at no time exceeded a hundred beats, it came up gradually to that point, remained there about twenty-four hours, and daily receded quietly to a healthy standard. We can only account for the phenomena, by the quantity of blood lost in the beginning, being the probable amount requisite for a quiet course, and a happy termination of the case.

